# PEARR tool training and implementation: building awareness of violence and human trafficking in a hospital system

**DOI:** 10.3389/fmed.2024.1311584

**Published:** 2024-05-09

**Authors:** Dominique Roe-Sepowitz, Kristen Bracy, Holly Gibbs, Rae Lynn Stafford, Brooke Bernardin, Hanni Stoklosa

**Affiliations:** ^1^School of Social Work, Arizona State University, Phoenix, AZ, United States; ^2^CommonSpirit Health, Chicago, IL, United States; ^3^Department of Social and Behavioral Sciences, Harvard T. H. Chan School of Public Health, Boston, MA, United States; ^4^Department of Emergency Medicine, Brigham and Women’s Hospital, Harvard Medical School, Boston, MA, United States; ^5^HEAL Trafficking, Long Beach, CA, United States

**Keywords:** human trafficking, trauma-informed care, PEARR tool, violence, abuse

## Abstract

**Introduction:**

Health professionals have an opportunity to assist patients who are experiencing many types of violence, including human trafficking; however, current approaches are often not person-centered. The Provide privacy, Educate, Ask, Respect and Respond (PEARR) Tool, a recognized screening tool in the U.S., is a structured conversation guide for health professionals on how to provide trauma-sensitive assistance to patients who may be experiencing such violence, including human trafficking. This is the first study to evaluate the PEARR Tool and its use in hospital settings.

**Methods:**

A U.S.-based health system adopted the PEARR Tool as part of its Abuse, Neglect, and Violence policy and procedure. To support successful adoption, the health system also developed educational modules on human trafficking and trauma-informed approaches to patient care, including a module on the PEARR steps. In October 2020 and June 2021, a voluntary “PEARR Tool Training and Implementation Survey” was distributed to emergency department staff in three hospitals. The survey consisted of 22 questions: eight demographic and occupation related questions; five questions related to the education provided to staff; and, nine questions related to the use of the PEARR Tool in identifying and assisting patients.

**Results:**

The overall findings demonstrate a general increase in awareness about the prevalence of human trafficking, as well as a significant increase in awareness about the implementation of the PEARR Tool. However, the findings demonstrate that most respondents were not utilizing the PEARR Tool between October 2020 and June 2021. Most reported that the reason for this was because they had not suspected any of their patients to be victims of abuse, neglect, or violence, including human trafficking. Of those that had utilized the PEARR Tool, there was a marked increase in staff that reported its usefulness and ease of access when caring for patients.

**Discussion:**

The COVID-19 pandemic posed many challenges during this study, including delays in staff education, changes in education format and delivery, and strains on staff. Initial data regarding the use of the PEARR Tool is promising; and additional research is recommended.

## 1 Introduction

Human trafficking, or trafficking in persons, is an abhorrent type of violence that impacts every region of the world (1). The United Nations General Assembly defines trafficking in persons as “the recruitment, transportation, transfer, harboring or receipt of persons, by means of the threat or use of force or other forms of coercion, of abduction, of fraud, of deception, of the abuse of power or of a position of vulnerability or of the giving or receiving of payments or benefits to achieve the consent of a person having control over another person, for the purpose of exploitation. This includes, at a minimum, the exploitation of the prostitution of others or other forms of sexual exploitation, forced labor or services, slavery or practices similar to slavery, servitude or the removal of organs” (2, p. 2).

In 2022, it was estimated that there are nearly 50 million people experiencing human trafficking globally (3). In a 2023 scoping review, the World Health Organization reported that the “health effects of trafficking and the health needs of trafficked individuals and trafficking survivors are well documented and urgent” (4, p. ii). The report underscored that health professionals “may be some of the few public servants to meet individuals while they are being trafficked” and that health systems have “both a responsibility and an opportunity to promote and protect the health and other rights of trafficked people” (4, p. vi). However, it also highlighted that there are a limited number of and limited use of validated screening tools; the report calls for Member States to “promote the incorporation and evaluation of standardized screening tools to identify trafficked individuals” (4, p. viii).

As many as 68% of people who experience human trafficking will have contact with a health care provider, which represents an opportunity for intervention. Yet, a minority of providers report confidence in their ability to identify patients who are experiencing human trafficking (5). Moreover, patients are frequently reluctant to disclose their status to health care providers due to fear of retaliation from their trafficker, fear of arrest or deportation, lack of privacy or confidentiality, language barriers, lack of trust, provider discrimination, and uncertainty about the benefits of disclosure (6, 7). These factors highlight the need for a non-disclosure focused, systems-based, trauma-informed approach to screening and assessment in which multi-sector resources are offered to patients in a safe environment, which empowers patients to share experiences and/or request assistance if they choose to do so (8, 9).

### 1.1 PEARR tool

The Provide privacy, Educate, Ask, Respect and Respond (PEARR) Tool is a recognized screening tool in the United States for various types of violence, particularly human trafficking, for patients in health care settings (10–12); however, it is not yet validated. This is the first study to investigate the PEARR Tool and its use in hospital settings. The PEARR Tool was first developed by Dignity Health, a U.S. nonprofit health system, in partnership with HEAL Trafficking (13) and Pacific Survivor Center (14), and was later adapted by CommonSpirit Health, also a U.S. nonprofit health system (15). The tool itself is a three-page document which includes a summary of the PEARR steps, an overview of risk factors for and indicators of various types of violence, and a summary of national victim assistance hotlines with an editable section to add and update information about local agencies (e.g., law enforcement, child welfare, adult protective services, and non-governmental agencies) (16).

The PEARR Tool is a structured conversation guide for health professionals on how to provide trauma-sensitive assistance to patients who may be experiencing abuse, neglect, or violence, such as human trafficking (16). It promotes an approach in which patients are educated about violence, using brochures if possible, before further screening is conducted (16). The goal is to have a normalizing conversation with patients in order to promote health, safety, and well-being, and to create a safe environment for affected patients to possibly share their own experiences and/or accept further services (16). The PEARR Tool includes double asterisks that indicate points at which this sensitive conversation with a patient may end, prompting additional steps such as mandated reporting (16).

The steps from page one of the PEARR Tool are provided below (16).

Provide privacy: Discuss sensitive topics alone and in a safe, private setting (ideally a private room with closed doors). If a companion refuses to be separated from the patient, this may be an indicator of abuse, neglect, or violence.** Strategies to speak with the patient alone: Suggest the need for a private exam. For virtual or telephonic visits, request that the patient moves to a private space but proceed with caution as the patient may not actually be alone.** Note: Companions are not appropriate interpreters, regardless of communication abilities. In order to ensure safety for the patient, use a professional interpreter per your facility’s policy.** Also, explain limits of confidentiality (e.g., mandated reporting requirements); however, do not discourage the patient from disclosing victimization. The patient should feel in control of disclosures. Mandated reporting includes your requirements to report concerns of abuse, neglect, or violence, as defined by applicable laws or regulations, to internal or external authorities or agencies, as described by laws and regulations.
Educate: Educate the patient in a manner that is nonjudgmental and normalizes sharing of the information. Example: “I educate many of my patients about [fill in the blank] because violence is common in our society, and violence has a big impact on our health, safety, and well-being.” Use a brochure or safety card to review information about abuse, neglect, or violence, such as human trafficking, and offer the brochure or card to the patient. Ideally, this brochure or card will include information about resources (e.g., local service providers, national hotlines). Example: “Here are some brochures to take with you in case this is ever an issue for you, or someone you know.” If the patient declines the materials, respect the patient’s decision.**.
Ask: Allow time for open discussion with the patient. Example: “Is there anything you’d like to share with me? Would you like to speak with [insert advocate/service provider] to receive additional information for you, or someone you know?”** If physically alone with the patient, and especially if you observe significant concerns (e.g., a high number or pattern of risk factors) or indicators of victimization, ASK about concerns. Example: “I’ve noticed [insert risk factor/indicator]. You do not have to share details with me, but I’d like to connect you with resources if you are in need of assistance.”** Note: Limit questions to only those needed to determine the patient’s safety, connect the patient with resources (e.g., trained victim advocates), and guide your work (e.g., perform a medical exam). Optional: If available and as appropriate, use an evidence-based tool to screen the patient for abuse, neglect, or violence.
Respect & Respond: If the patient denies victimization or declines assistance, respect the patient’s wishes.** If you still have concerns about the patient’s safety, offer the patient a discrete hotline card or other information about emergency services (e.g., a local shelter). Otherwise, if the patient accepts or requests assistance, arrange a personal introduction with a local victim advocate (see page 3) or assist the patient in calling a national hotline: Domestic Violence Hotline, 1-800-799-7233; Sexual Assault Hotline, 1-800-656-4673; Human Trafficking Hotline, 1-88-373-7888.**.
** Report safety concerns to appropriate personnel (e.g., a security officer), complete mandated reporting, and continue trauma-informed health services. Whenever possible, schedule follow-up appointments to continue building rapport with the patient and to monitor the patient’s health, safety, and well-being.

In 2019, Dignity Health (now known as CommonSpirit Health) adopted a system-wide Abuse, Neglect, and Violence policy and procedure, a key component of which was the PEARR Tool. To support successful adoption of the policy and procedure, this health system also developed educational modules on human trafficking and trauma-sensitive approaches to patient care, including a module on the PEARR steps. The purpose of this study was to evaluate the experiences of emergency department staff regarding implementation of the policy and procedure, particularly the PEARR Tool, and associated education in three hospitals in a large city in central California, U.S.

## 2 Methods

The educational modules on human trafficking and trauma-sensitive approaches to patient care, including the module on the PEARR steps, were originally designed for in-person delivery with discussion. The research team intended to provide pre- and post-surveys to staff at the start and end of the face-to-face training. However, in-person education was no longer possible as the coronavirus (COVID-19)-pandemic in 2019 caused system-wide restrictions on travel and in-person meetings. Therefore, the educational modules were saved as read-only PDFs, uploaded to an internal learning management system, and assigned to staff across the three hospitals on 1 March 2020, including approximately 300 staff members whose role included caring for patients in the emergency department.

The original deadline for staff to complete the education was 31 March 2020; however, as the pandemic continued, this deadline was extended to 30 September 2020. To help supplement this education, live “mini-trainings” were provided, as able. For example, short (e.g., 5-min) mini-trainings were provided at department huddles and during shift changes, and longer (e.g., 30-min) trainings were provided on department meeting calls. Attendance for these meetings was often low due to staff shortages and other COVID-related disruptions. Informational posters on possible signs of human trafficking among patients were also posted in staff breakrooms and other staff common areas.

To evaluate the implementation of the PEARR Tool, the research team provided a voluntary survey (see section 2.1) to emergency department staff in two waves, following the extended deadline to complete the education. Originally, the research team had intended to interview staff in-person, and to do so in several waves to measure pre-knowledge of human trafficking and victim response procedures (prior to education and implementation of the PEARR Tool) and ongoing knowledge of human trafficking and victim response procedures (after education and implementation of the PEARR Tool). This plan later changed to surveying staff in a lesser number of waves. These changes were due to various challenges, particularly those described above. This study received research approval from both Arizona State University and Dignity Health institutional review boards.

### 2.1 Instrument

In October 2020 and June 2021, the voluntary “PEARR Tool Training and Implementation Survey” was distributed to emergency department staff in three Dignity Health hospitals based in central California, U.S. (i.e., “Hospitals A, B, and C”). The paper-and-pencil survey was one-page front and back and consisted of 22 questions: eight demographic and occupational related questions, five questions related to education provided to hospital staff about human trafficking, and nine questions related to the use of the PEARR Tool and electronic health record system (Cerner) in identifying and assisting patients experiencing abuse, neglect, or violence, such as human trafficking.

This survey was not a standardized instrument, and there were no plans to validate it or to create subscales. This was strictly a tool used to collect information from staff about ongoing knowledge of human trafficking and their experiences with the implementation of the policy and procedure, particularly the PEARR Tool, and associated education. Additional research is recommended using validated instruments to further evaluate the PEARR Tool and its use in health care settings.

The questions and answer options included the following:

- How common do you believe human trafficking (labor and sex trafficking) is in your community? Very Common, Common, or Not Common?
- How common do you believe an experience of human trafficking is among the patients you serve? Very Common, Common, or Not Common?
- How common do you believe human trafficking is in the United States as a whole? Answer options? Very Common, Common, or Not Common?
- Have you received training on human trafficking in the past? Yes or No?
- Do you believe that there are adequate resources and support at your workplace to provide appropriate care and services to victims of human trafficking? Yes or No?
- Has the PEARR Tool been put into place at your workplace for identifying and serving victims of human trafficking? Yes or No?
- Have you used the PEARR Tool in your workplace? Yes or No?
- If no, what are the reasons you have not used the PEARR Tool yet? [see section 3.3 for answers].
- Was the PEARR tool useful in communication with your patient and addressing their needs? Yes, No, or Not Applicable?
- Were you able to access the PEARR Tool guidelines in Cerner? Yes or No?
- Did you arrange for a private setting with each of these patients in order to provide education or offer victim assistance? Yes, No, or I have not flagged any patients in Cerner?
- Did you provide each of these patients with education about any types of violence that were of concern, and did you use brochures or other materials to assist with this conversation?
  - Yes, I provided education using brochures and materials.
  - Yes, I provided education but did not use brochures or materials.
  - No, or I have not flagged any patients in Cerner.
- Did you assist any of these patients with a warm referral to a community agency (i.e., a personal introduction—either by phone or in-person)? Yes, No, or I have not flagged any patients in Cerner?
- Did you offer the patients any resources that feature information (e.g., crisis hotlines) to assist in the event of an emergency especially if this person declined to be connected with community agencies? Yes, No, or I have not flagged any patients in Cerner?

The responses from the two data collection points (October 2020 and June 2021) were compared using chi-square analysis to assess for changes over time in respondent answers.

## 3 Results

### 3.1 Participants

Between the two data collection dates in October 2020 and June 2021, 179 hospital staff completed the voluntary “PEARR Tool Training and Implementation Survey.” A total of 75 staff completed the first wave of the survey in October 2020, and a total of 104 staff completed the final wave in June 2021. See Table 1 for a summary of demographic and occupational-related information.

**Table 1:** Summary of demographic and occupational related information of respondents.

Demographics and other descriptors of respondents	First wave (October 2020)	Second wave (June 2021)
**Primary hospital location**		
Hospital A	56	89
Hospital B	15	15
Hospital C	4	0
Total	75	104
**Gender identity**		
Female	59	71
Male	14	29
Transgender female	1	1
Transgender male	0	0
Gender variant/non-conforming	1	2
Other	0	0
Total	75	103 (1 did not answer)
**Ethnicity**		
African American	1	3
American Indian/Alaska Native	0	3
Asian/Pacific Islander	10	11
Biracial/Multiracial	5	7
Caucasian	28	44
Hispanic/Latino(a)	28	35
Other	1	1
Total	73 (2 did not answer)	104
**Highest level of education**		
High school diploma		1
Some college	2	8
Associate’s degree	27	41
Bachelor’s degree	31	36
Master’s degree	15	13
Professional degree/doctorate		2
Total	75	101 (3 did not answer)
**Credentials (Note: The survey included options to select credentials and roles, as shown below)**		
RN (i.e., Registered Nurse)	60	75
LVN (i.e., Licensed Vocational Nurse)	3	6
SUN (i.e., Substance Use Navigator)	1	1
NSM (i.e., Nurse Shift Manager)	4	1
CAN (i.e., Certified Nursing Assistant; should have been listed as CNA on the survey)	0	0
ED Tech (i.e., Emergency Department Technician)	0	11
Clinical Social Worker	8	7
Chaplain	1	4
Total	77 (multiple responses allowed)	105 (multiple responses allowed)

The majority of the respondents identified in some capacity as nursing staff (*n* = 132, 73.7%) with titles such as ED Nurse, ED Quality RN, ED RN, ED RW, EN RN, ER Nurse, ER RN, MICN (i.e., Mobile Intensive Care Nurse), MICN RN, MICN/RN, NSM, Nurse Shift Manager, Nursing, Quick Look ED, Registered Nurse, Registered Nurse in EP, RN, RN ED, RN NSM, RN Staff, and Travel RN.

Other respondents identified as

- Clinical Social Worker, Medical Social Worker, or Linkage Care Specialist (*n* = 17, 19.5%),
- ED Tech, ER Tech, or MT (Medical Tech) (*n* = 11, 6.1%),
- LVN (*n* = 7, 3.9%),
- Chaplain or Staff Chaplain (*n* = 5, 2.8%),
- Substance Use Navigator (*n* = 2, 1.1%),
- Director of Emergency Services (*n* = 1, 0.6%),
- Pharmacy Tech Med History (*n* = 1, 0.6%), and
- Three (1.7%) respondents did not provide this information.

Respondents’ years of experience in the health care field ranged from 1 year to 43 years, with an average of 11.7 years (*SD* = 8.64) and a combined total of 2,020 years of experience in the health care field. Respondents’ years of experience in their given job position ranged from zero to 32 years, with an average of 6.6 years (*SD* = 5.64) in their given job positions.

The responses from two data collection points (October 2020 and June 2021) were compared using chi-square analysis to assess for changes over time in respondent answers (see sections 3.2 and 3.3).

### 3.2 Perception of prevalence of human trafficking

#### 3.2.1 Perception of prevalence of human trafficking in community

**Figure 1:**
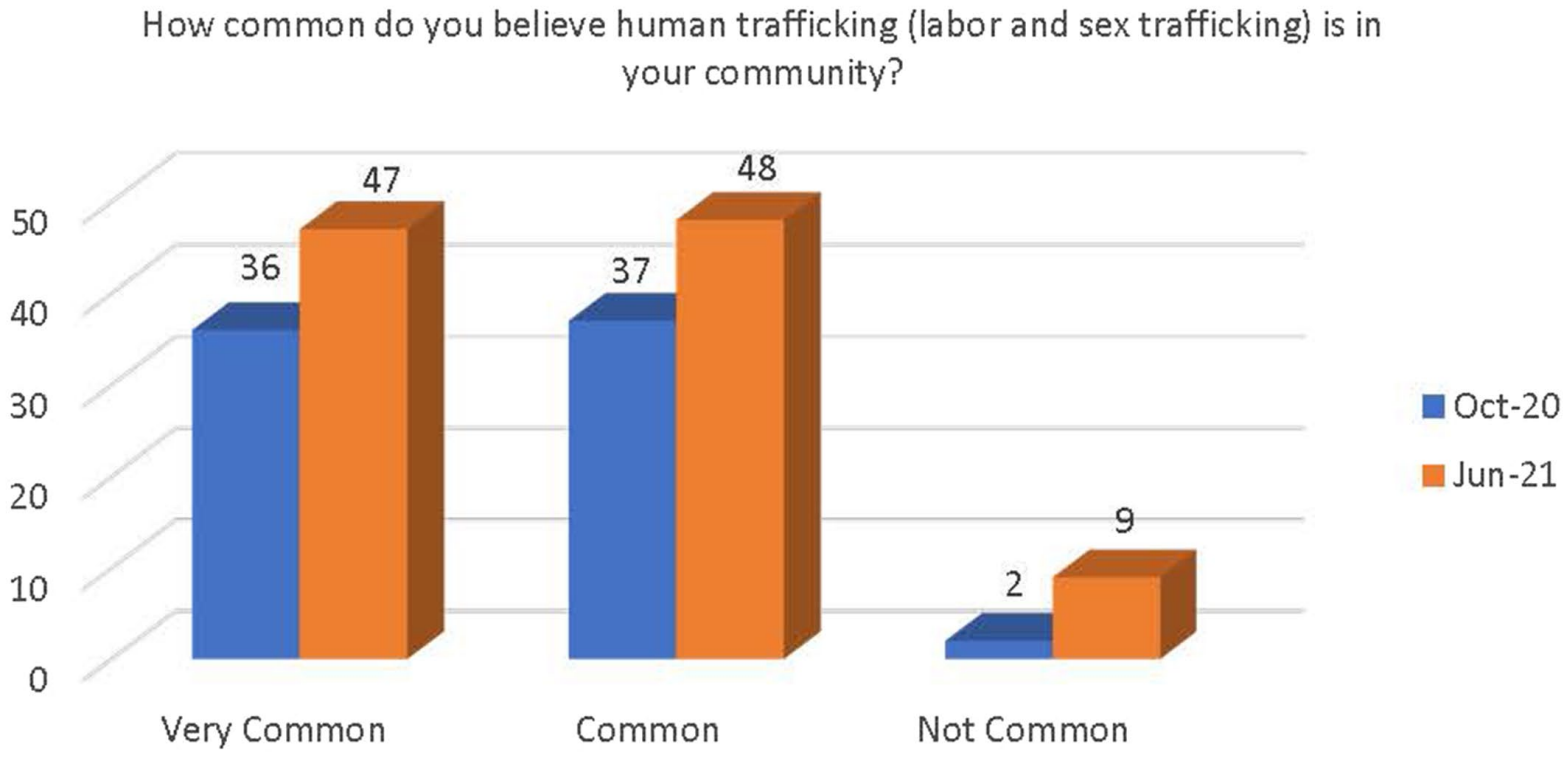
Change in belief on prevalence of human trafficking in the community.

Respondents were asked how common they feel the issue of human trafficking (labor and sex trafficking) is in their community. The options were very common, common, or not very common. Although not significant, there was a demonstrated increase in respondents’ belief that human trafficking is very common or common in their community (see Figure 1).

#### 3.2.2 Perception of prevalence of human trafficking among patients

**Figure 2:**
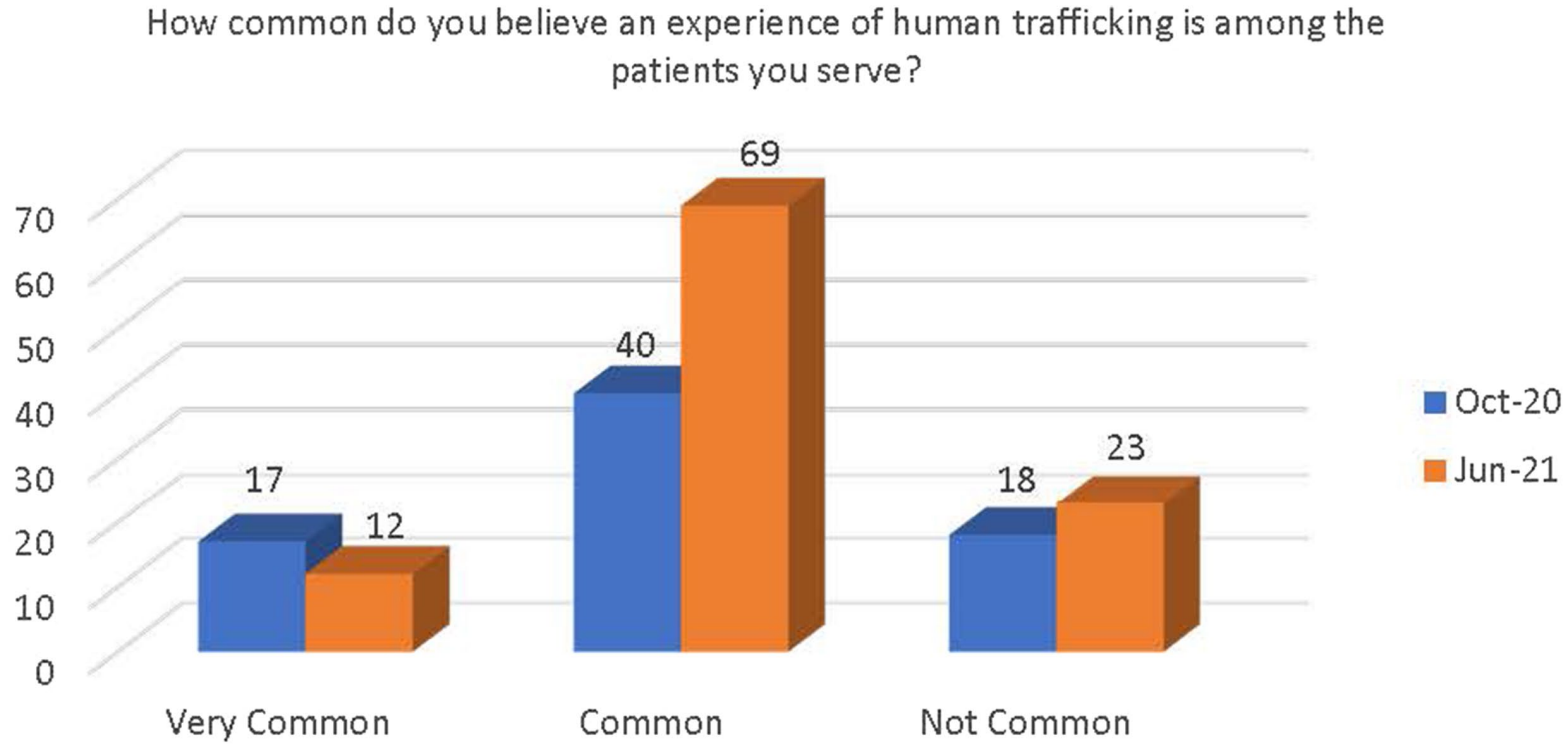
Change in belief on prevalence of human trafficking among patients.

Respondents were asked how common they feel an experience of human trafficking is among the patients they serve. Although not significant, there was a demonstrated increase in respondents’ belief that an experience of human trafficking is common among their patients. There was also a slight decrease in the belief that human trafficking is very common and a slight increase in the belief that human trafficking is not very common among their patients (see Figure 2).

#### 3.2.3 Perception of prevalence of human trafficking in the United States

**Figure 3:**
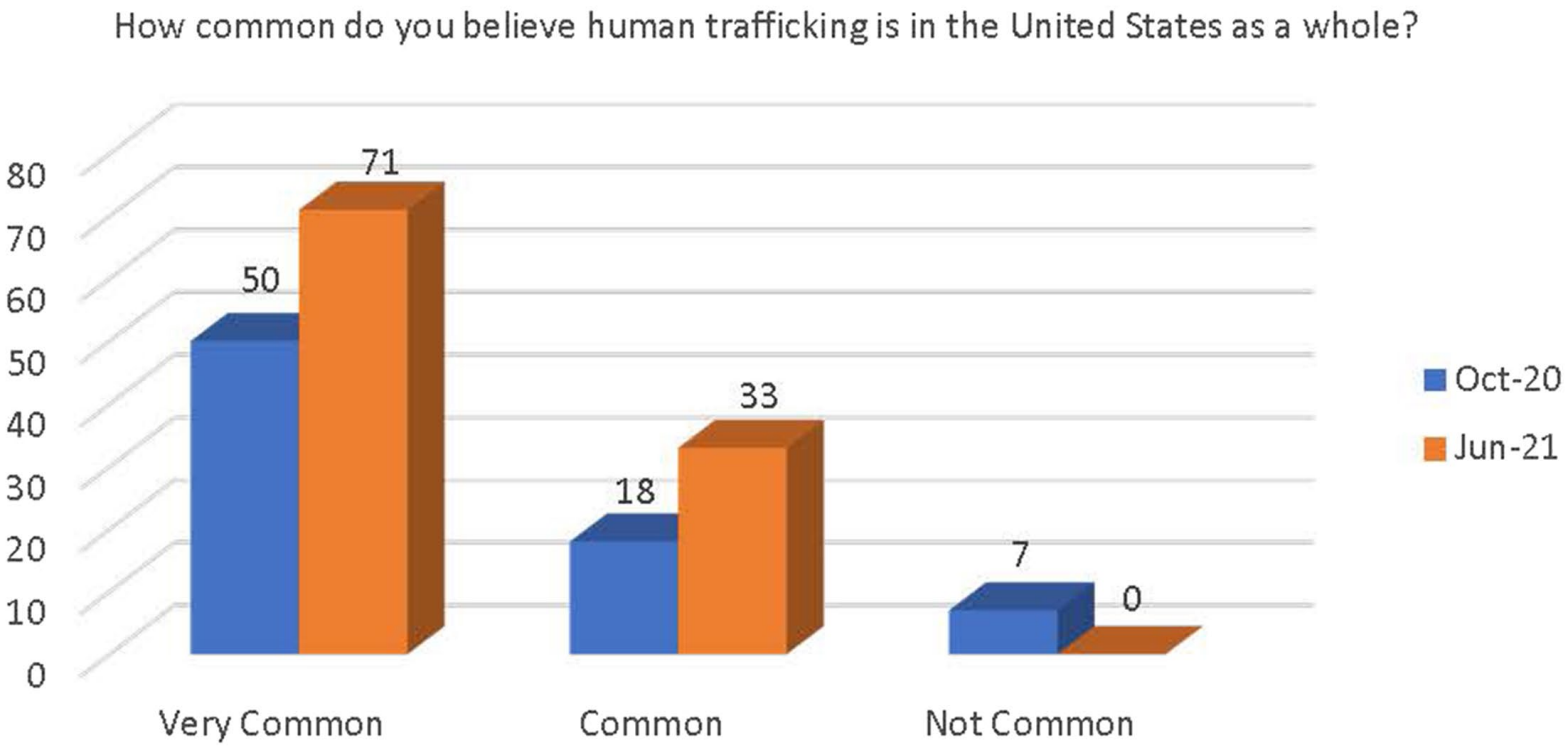
Change in belief on prevalence of human trafficking in the United States as a whole.

Respondents were asked how common they feel the issue of human trafficking is in the United States as a whole. Although not significant, there was a demonstrated increase in respondents’ belief that human trafficking in the United States is very common or common. There was also a decrease in respondents’ belief that human trafficking in the United States is not very common (see Figure 3).

### 3.3 Training and PEARR tool implementation

**Figure 4:**
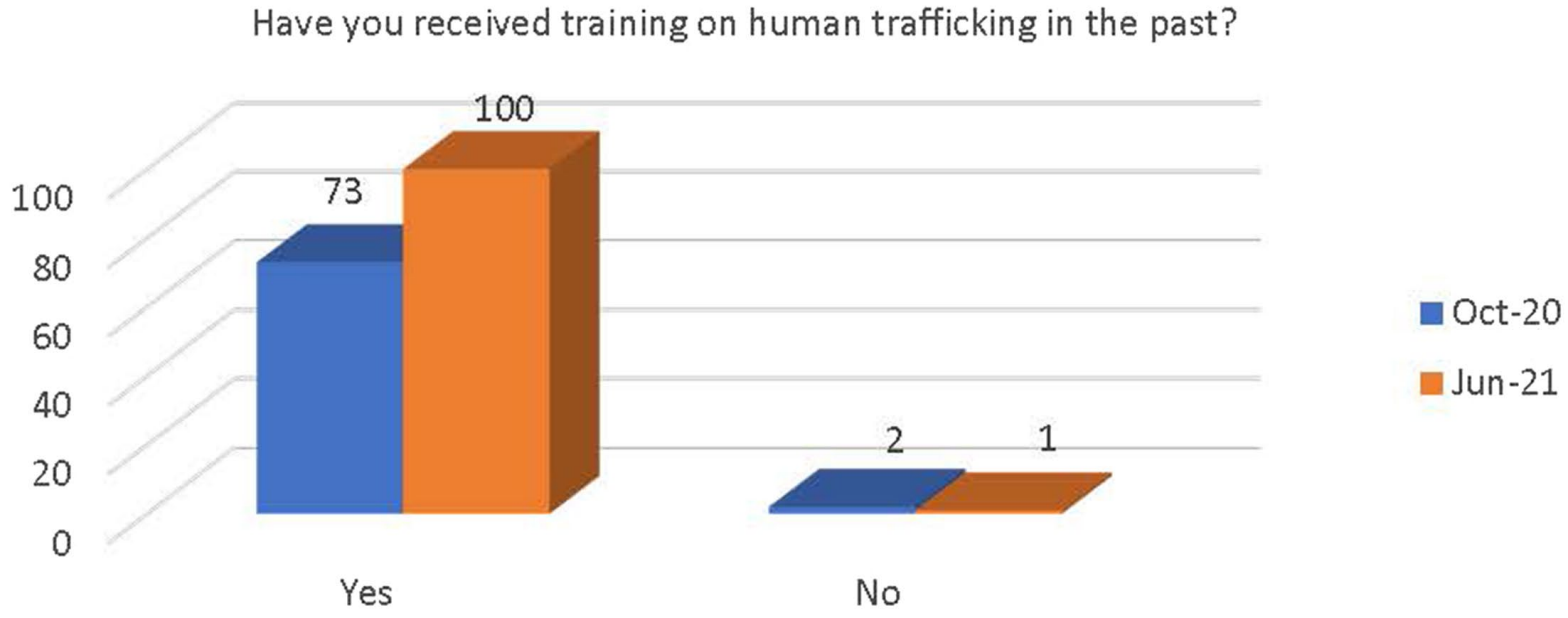
Number of respondents who have received human trafficking training.

**Figure 5:**
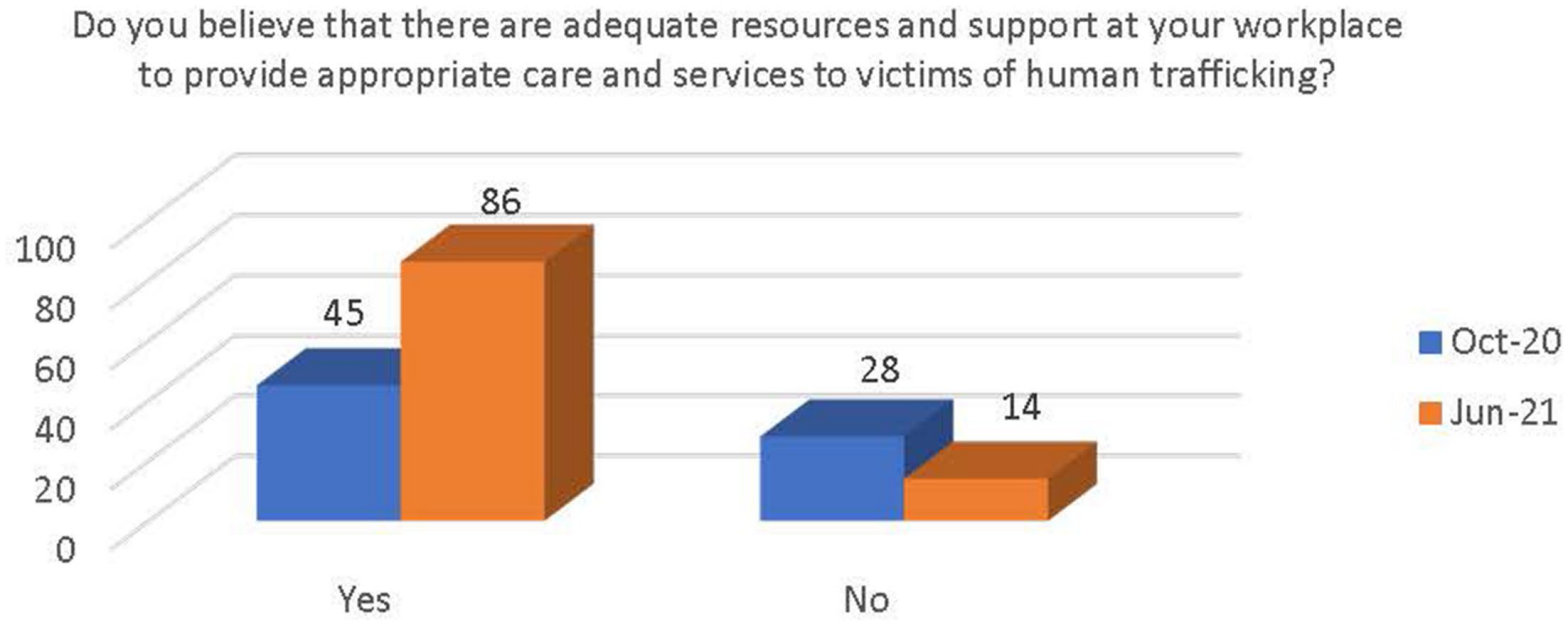
Respondents’ belief of adequate resources and support for human trafficking victims.

**Figure 6:**
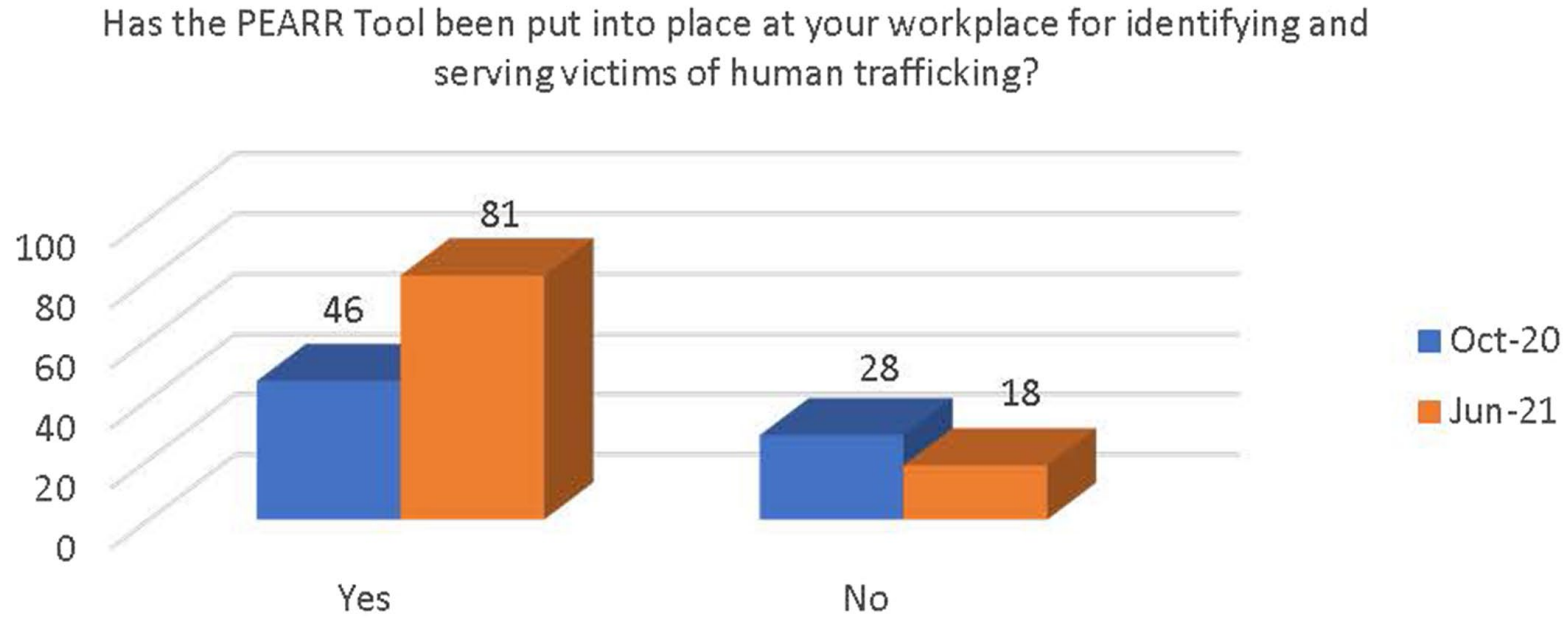
Respondents’ awareness about implementation of the PEARR Tool.

Respondents were asked if they had received training on human trafficking in the past. Most reported that they had received training (see Figure 4). There was a significant increase in the number of respondents who believe that there are adequate resources and support available at their workplace to provide appropriate care and services to victims of human trafficking [x^2^(1, *N* = 173) = 13.62, *p* < 0.000; see Figure 5]. There was also a significant increase in awareness that the PEARR Tool had been implemented at the respondents’ workplace [x^2^(1, *N* = 173) = 8.38, *p* < 0.004; see Figure 6].

**Figure 7:**
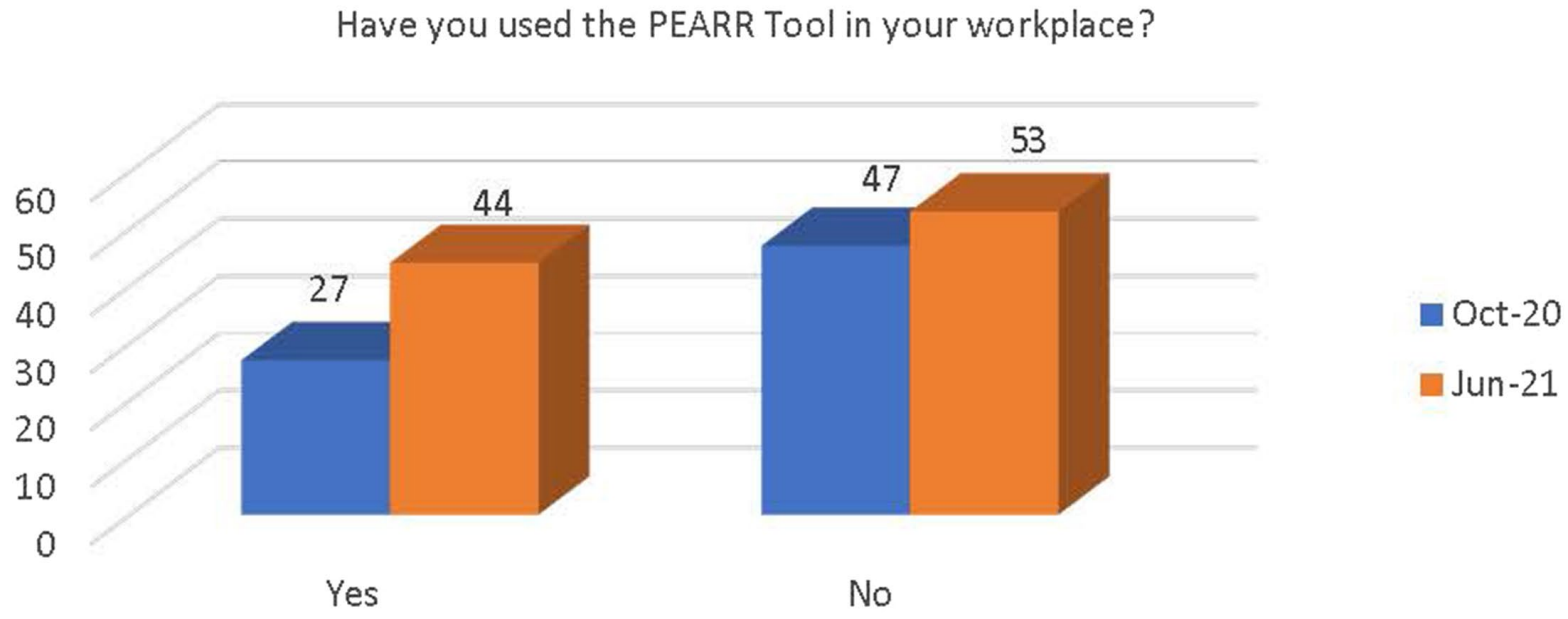
Respondents’ use of the PEARR Tool in their workplace.

Respondents were asked if they had begun utilizing the PEARR Tool in their workplace. There was an increase in respondents reporting that they had begun using the PEARR Tool; however, there was also an increase in respondents reporting that they had not. More respondents reported that they had not begun using the PEARR Tool (see Figure 7). The latter respondents were asked to provide reasons as to why they had not begun utilizing the PEARR Tool. The options were as follows:

- I have not suspected any of my patients to be victims of abuse/neglect/violence/human trafficking.
- I did not understand how to use the PEARR Tool.
- I could not find the PEARR Tool when I was with a patient.
- I did not have access to the materials (brochures, phone numbers) that I needed to share with the patient.
- I could not find the PEARR Tool guidelines in Cerner.

**Figure 8:**
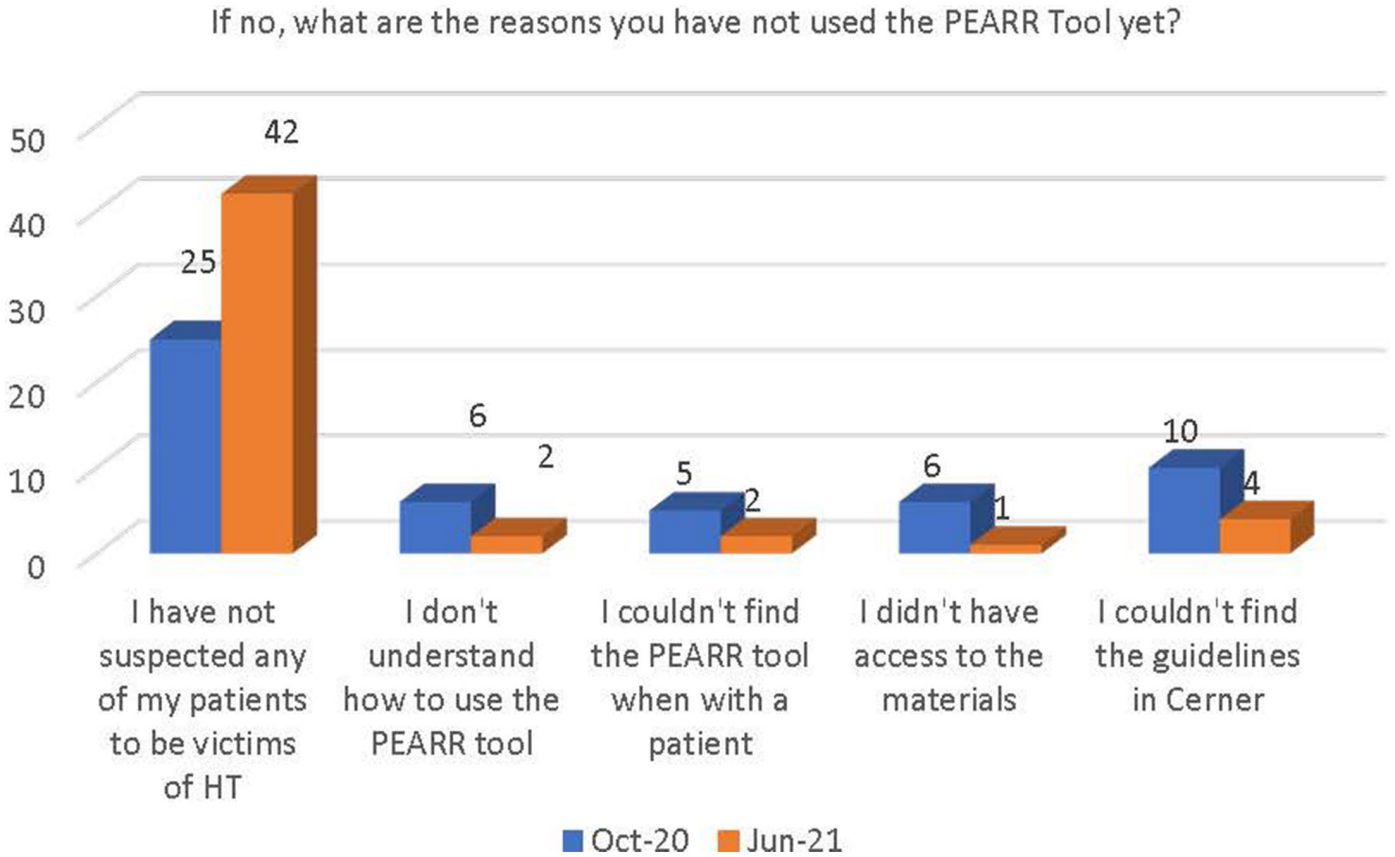
Why respondents had not yet utilized the PEARR Tool.

Most respondents reported that the reason they had not utilized the PEARR Tool in their workplace was because they had not suspected any patients to be victims of abuse, neglect, or violence, including human trafficking. Very few reported that they did not understand how to use the PEARR Tool, could not find the PEARR Tool when they were with a patient, did not have access to the materials when they were with the patient, or that they could not find the PEARR Tool guidelines in the electronic health record system (Cerner); these responses also decreased in June 2021 (see Figure 8).

**Figure 9:**
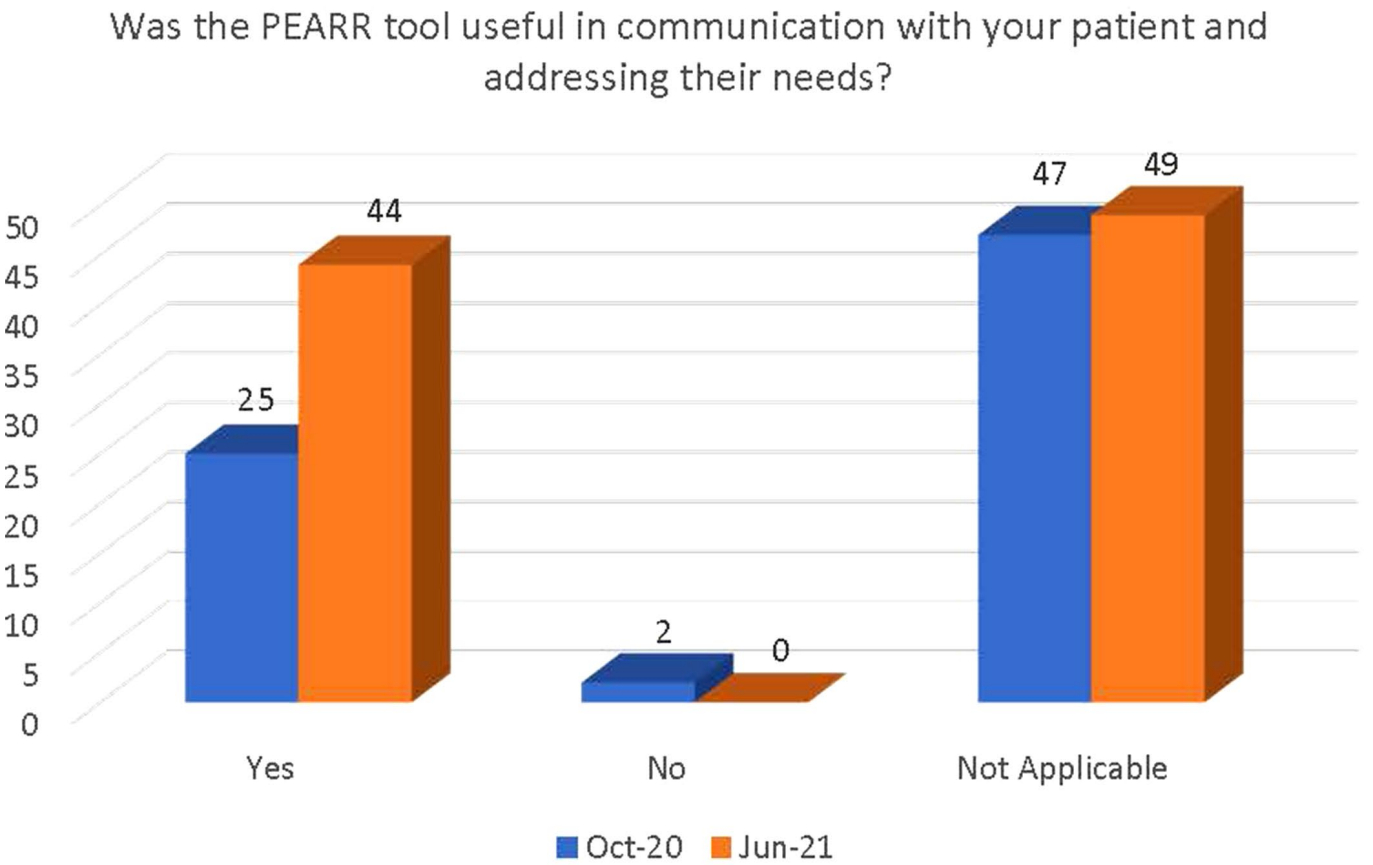
Perceived usefulness of the PEARR Tool in communication with patient.

**Figure 10:**
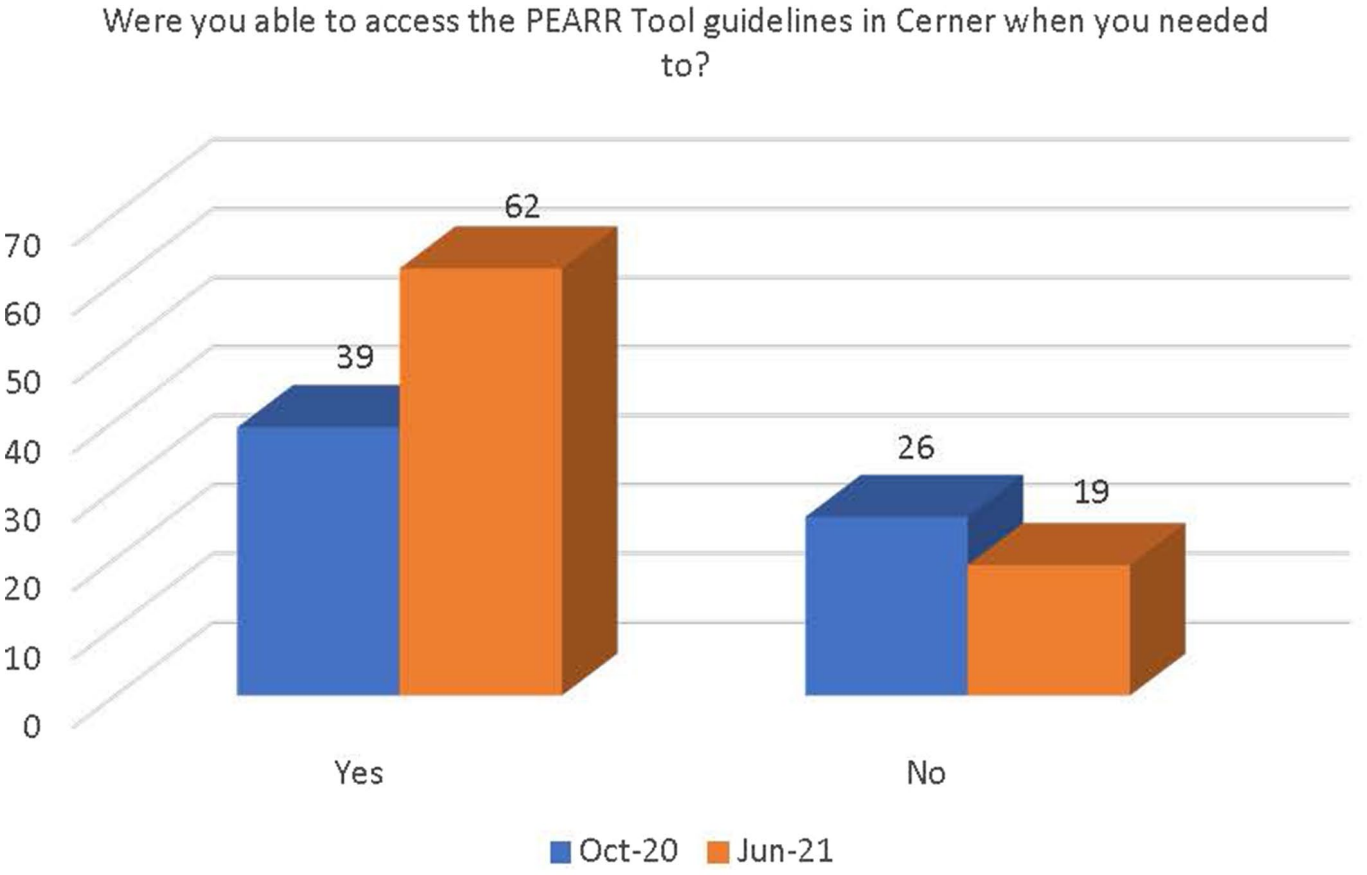
Respondents’ ease of access to the PEARR Tool guidelines in Cerner.

The final section of the survey inquired about the access and ease of use of the PEARR Tool and if the respondent took the appropriate steps (as outlined by the tool) when necessary. Most respondents in both the October 2020 and June 2021 data collection periods selected the following options: Not applicable or I have not flagged any patients in Cerner. Of those that had utilized the PEARR Tool, there was a demonstrated increase in reported usefulness of the tool and ability to access the PEARR Tool guidelines in the electronic health record system (Cerner; see Figures 9, 10).

**Figure 11:**
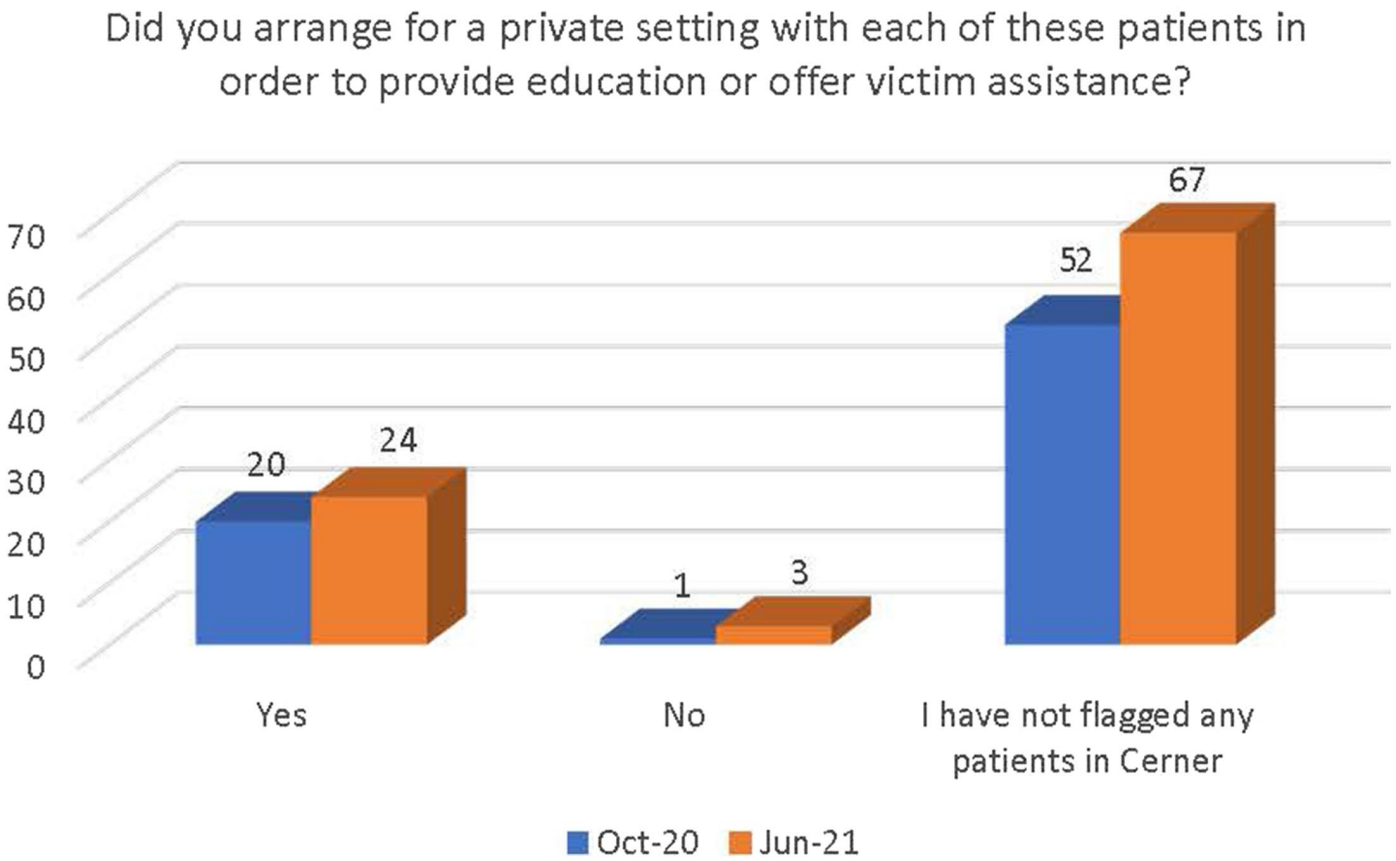
Number of respondents who provided a private setting for the patient.

**Figure 12:**
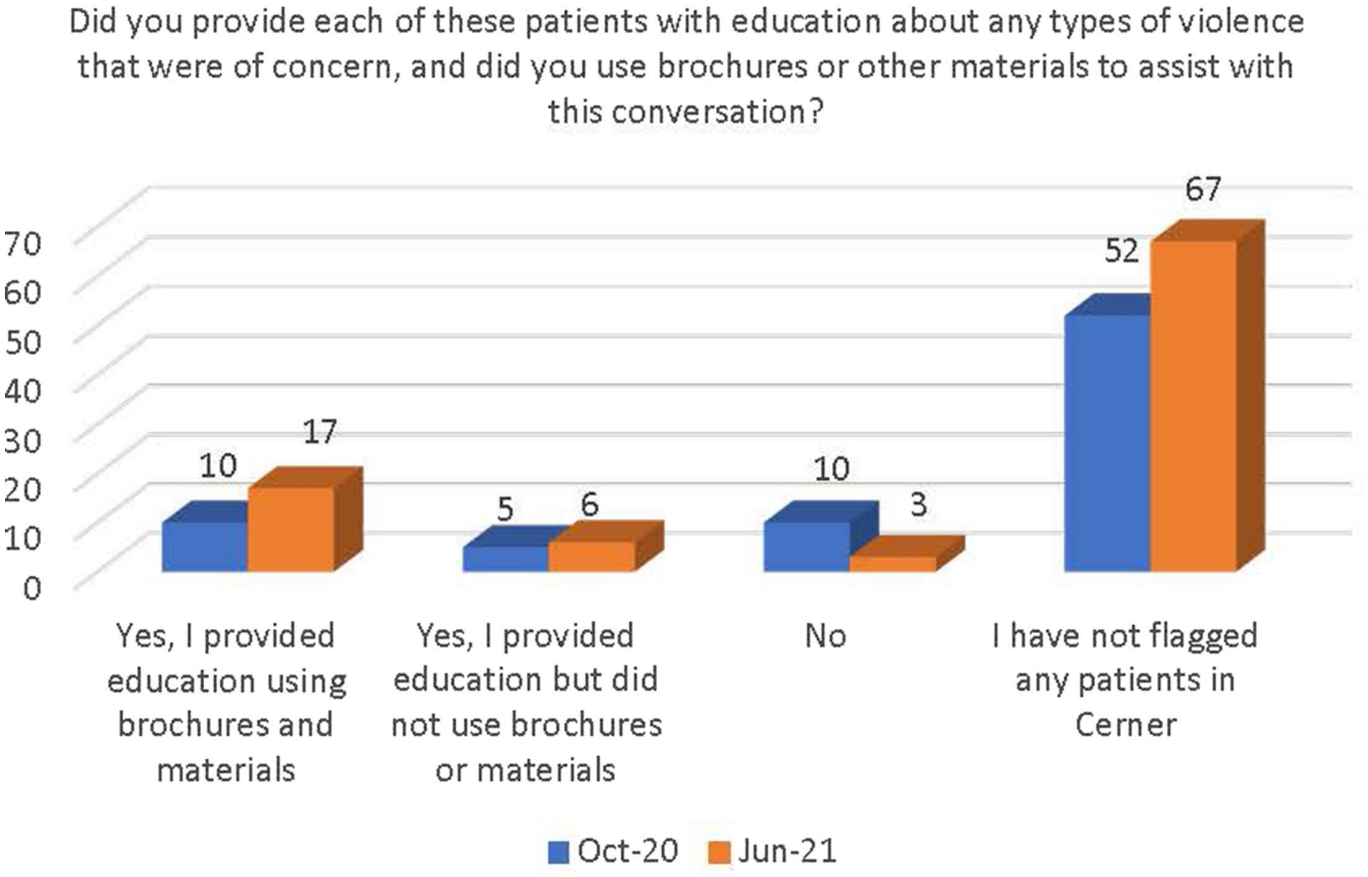
Number of respondents who provided patients with education and used materials.

**Figure 13:**
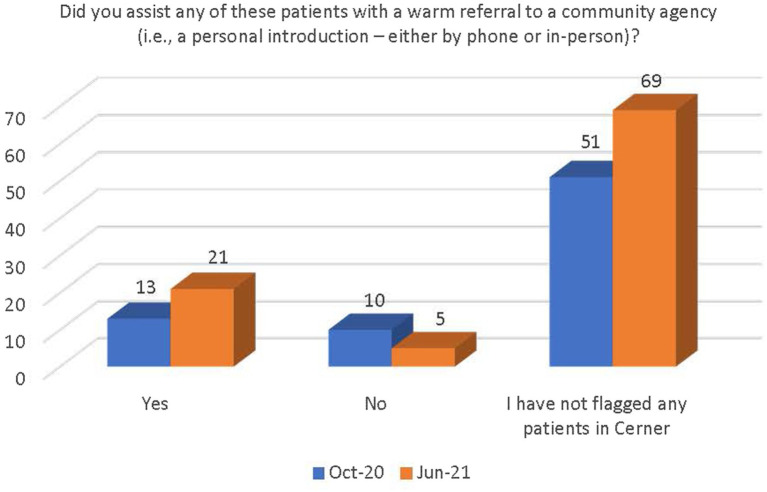
Number of respondents who provided a referral for services to a community agency.

**Figure 14:**
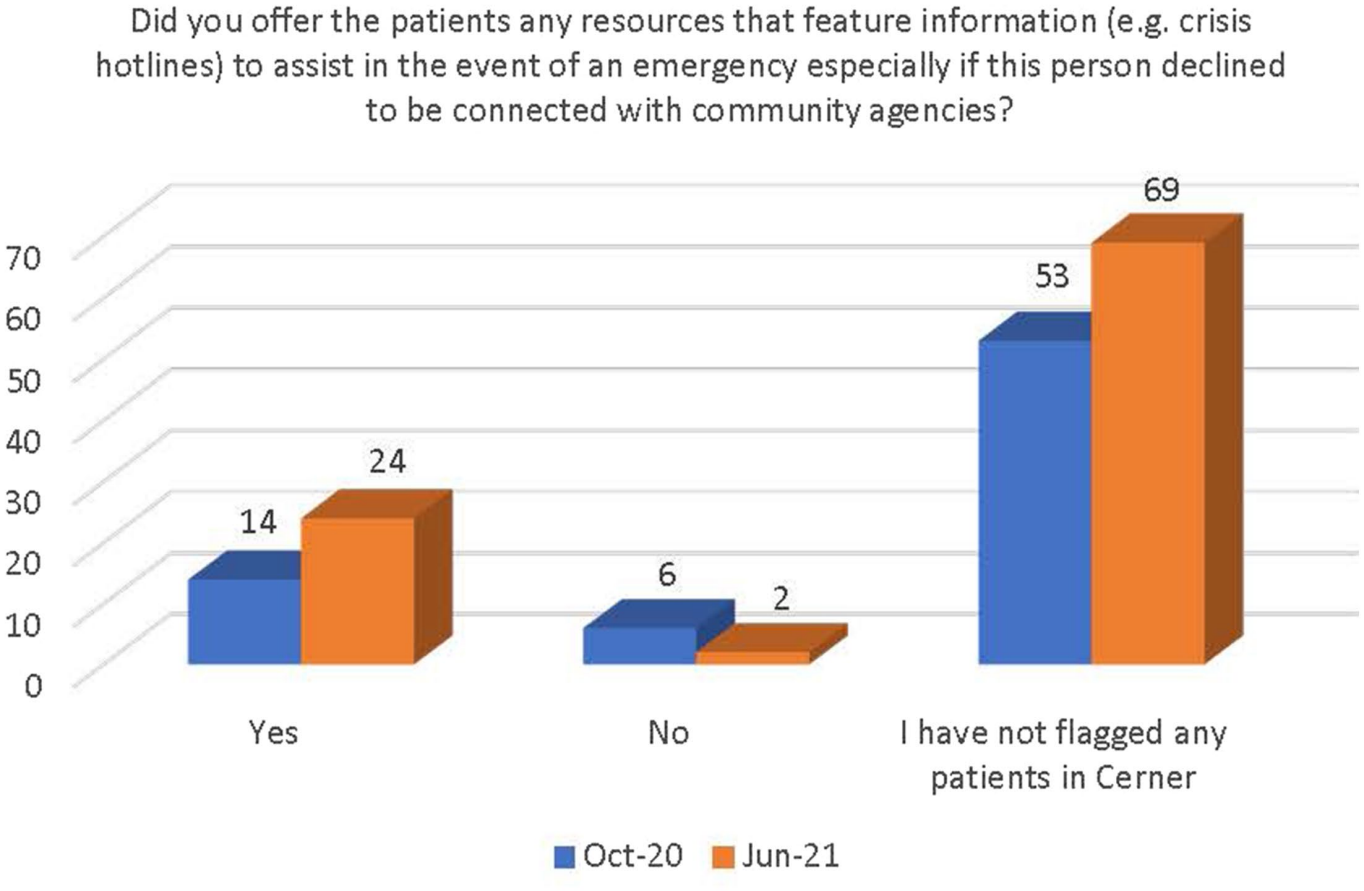
Number of respondents that offered patients resources to assist in an emergency.

There was a general increase in the number of respondents who followed the guidance outlined in the PEARR Tool, including providing privacy for the patient, educating the patient about violence and resources (with or without the use of brochures or other materials to support this conversation), asking the patient about concerns and assisting with a warm referral to a community agency, if/as requested by the patient, and respecting the patient’s decisions, as appropriate. The last step includes offering hotline cards for agencies that can assist in the event of an emergency, especially if the patient declines to be connected with community agencies (see Figures 11–14).

## 4 Discussion

The findings of the voluntary “PEARR Tool Training and Implementation Survey” demonstrate that most respondents were not utilizing the PEARR Tool between October 2020 and June 2021. Most reported this was because they had not suspected any patients to be experiencing abuse, neglect, or violence, including human trafficking. It is not possible to determine if this lack of identification was a result of people who were experiencing violence in the community avoiding utilization of the health system’s emergency departments or due to missed recognitions on the part of staff. However, it is known that health care staff were overwhelmed during this time by surges in hospital and emergency department admissions and that such conditions can cause other urgent situations, such as violence intervention efforts, to be overlooked and the opportunity to offer such support to patients missed (17).

Research exploring the effects of the COVID-19 pandemic on health care provision suggests that both emergency department utilization and the screening/identification capacity of health professionals were negatively impacted. Emergency department visits decreased worldwide during the pandemic, even for serious conditions such as heart failure and stroke (18). Moreover, during the COVID-19 pandemic, providers in the emergency department experienced drastic changes in the way that they could provide care and build trust with their patients. Providers reported limiting the amount of time they spent in patient rooms and perceived that personal protective equipment obscuring their facial expressions added to patient discomfort (19). These factors make providers less likely to engage in screening and patient education that they deem nonessential, and create barriers to discussing sensitive topics like domestic violence, sexual violence, and human trafficking. Indeed, general practitioners across 33 countries reported that they screened patients for domestic violence less often during the COVID-19 pandemic compared to before the pandemic and that patient disclosures of domestic violence decreased (20). Similar factors might have limited the use of the PEARR Tool in this study.

Ongoing state-of-the-art education and skills building among staff about how to identify vulnerabilities to and indicators of abuse, neglect, and violence, including human trafficking, may result in an increase in identifications and use of the PEARR Tool. Although the majority of staff in this study had completed the education by the extended deadline, the modules were strictly available as read-only PDFs. Given COVID-related limitations in delivering education in-person using adult learning principles, it is possible that staff’s mental models of what trafficked individuals look like may not have matched the breadth of trafficking experiences with which they may have had contact (21).

This health system now has an interactive educational module on human trafficking in its learning management system; the module features videos of diverse survivors of labor trafficking and sex trafficking sharing their experiences and encounters with health care. This module is also available for live education (in-person or virtual) with staff and community partners. It provides basic information on human trafficking, including possible red flags in patient care settings. The learning objectives include defining human trafficking, recognizing misconceptions often associated with this type of violence, identifying risk factors that can make a person vulnerable to exploitation, and taking action to appropriately assist patients who may be affected by human trafficking or other types of violence. This health system promotes educating all staff about this topic, including non-clinical personnel who may observe concerns involving patients while working in the hallways, waiting areas, or parking lots.

Although few respondents in this study reported that they did not understand how to use the PEARR Tool, this response indicates the importance of educating staff about the tool as well. In addition to having educational modules on human trafficking and trauma-informed approaches to patient care, this health system also has an interactive educational module in its internal learning management system on the PEARR steps. This module provides an in-depth description of the PEARR steps with the following objectives: Identify patients at risk of abuse, neglect, or violence, offer assistance to patients using the PEARR steps, and access brochures and safety cards that can support this process. This module is also available for live education with staff and community partners.

Although few respondents reported that they could not find the PEARR Tool when they were with a patient, or that they did not have access to the materials that they needed to share with the patient, this represents a potential significant barrier to the use of this tool. The tool was designed with the PEARR acronym in order to make the steps easier to remember. The goal is for staff to complete the education on the PEARR steps and to have the three-page PEARR Tool handy as a reference guide, but not to rely on the guide when walking through the five steps with patients. This health system has an additional educational module that features 10 health care scenarios shared by labor trafficking and sex trafficking survivors, all of whom were offered payment for their participation as subject matter expert consultants. This module builds on the content provided in the previous modules and is meant to be delivered live to a multidisciplinary audience who will then recall the PEARR steps and other concepts covered in previous modules while discussing questions for each scenario.

This health system also encourages each of its hospitals to implement multidisciplinary Task Force teams that help to socialize the Abuse, Neglect, and Violence policy and procedures, including the PEARR Tool and associated education and resources. This helps to keep staff informed on where to find the PEARR Tool and any brochures or safety cards that can support with patient conversations. This health system also distributes staff badge buddies with a summary of the PEARR steps for quick reference. All of these efforts help to minimize barriers to staff finding information and resources when needed. Last, this health system implemented changes in its electronic health record system (Cerner) to align with the policy and procedures, including the PEARR steps. Many of these changes were delayed due to COVID-related setbacks; this created some confusion and helps to explain why some respondents reported that they could not find the PEARR Tool guidelines in Cerner.

Limitations of this study include a response rate among staff of 25%–35%, and the characteristics of non-responders is not known, thereby limiting the generalizability of the results. Furthermore, although responses from the October 2020 and June 2021 surveys were compared, it is unknown the amount of overlap between the two samples, and therefore the extent to which the results can speak to true change.

The data demonstrated that respondents generally believe that human trafficking is a concern in the community. However, the data also demonstrated that, between October 2020 and June 2021, less staff believed human trafficking was very common among patients. There was an increase in staff that believed human trafficking was common, and a slight increase in staff who believed human trafficking was not very common among patients. It is not possible to determine the impact that the COVID-19 pandemic might have had on these changing beliefs among staff. Ongoing data collection on the number of patients with documented concerns of abuse, neglect, or violence, such as human trafficking, may provide a more accurate picture of the prevalence and concerns of such violence among patients, and would be beneficial for heightened education and other programmatic improvement opportunities.

There was a significant increase between October 2020 and June 2021 in the number of staff who were (1) aware of the implementation of the PEARR Tool and (2) who felt that there was adequate support and resources available to them to provide assistance to patients affected by human trafficking. This is a positive indicator of the ongoing efforts by this health system to educate staff about human trafficking and to equip staff with useful tools and resources for assisting patients. However, most staff reported that they had not utilized the PEARR Tool at both the October 2020 and June 2021 data collection periods. Of those that had utilized the PEARR Tool, there was a marked increase in staff that reported its usefulness and ease of access. From October 2020 to June 2021 there were slight increases in the number of staff that followed the guidance outlined in the PEARR Tool, including providing privacy for the patient, educating the patient about violence and resources (with or without the use of brochures or other materials to support this conversation), asking the patient about concerns and assisting with a warm referral to a community agency, if/as requested by the patient, and respecting the patient’s decisions, as appropriate. The last step includes offering hotline cards for agencies that can assist in the event of an emergency, especially if the patient declines to be connected with community agencies.

Additional research should be conducted to evaluate the effectiveness of the educational modules described here and other strategies to educate staff about human trafficking and how to provide trauma-sensitive assistance to patients. This should include efforts to further evaluate the PEARR Tool using validated instruments, including barriers to its use and its potential long-term impact. In summary, the findings from the voluntary PEARR Tool Training and Implementation Survey demonstrate a general increase in awareness among staff about the prevalence of human trafficking nationally, in the community, and among patients, as well as a significant increase in awareness about the implementation of the PEARR Tool. Of those that had utilized the PEARR Tool, there was a marked increase in staff that reported its usefulness and ease of access when caring for patients.

Although this study shows promising results regarding the use of the PEARR Tool, additional research is recommended, particularly when staff are not strained by a pandemic. Human-centered design approaches utilizing implementation science are recommended. This includes evaluating staff education on various types of abuse, neglect, and violence, including human trafficking, and the use of the PEARR Tool in intervention efforts. Health professionals must be educated on violence, including labor trafficking and sex trafficking; otherwise, they will not be equipped to identify patients who might benefit from the assistance provided via the PEARR Tool (22).

## Data availability statement

The original contributions presented in the study are included in the article/supplementary material, further inquiries can be directed to the corresponding author.

## Ethics statement

The studies involving humans were approved by Dignity Health and Arizona State University Institution Review Board. The studies were conducted in accordance with the local legislation and institutional requirements. The participants provided their written informed consent to participate in this study.

## Author contributions

DR-S: Writing – original draft. KB: Writing – original draft. HG: Writing – review & editing. RS: Writing – review & editing. BB: Writing – review & editing. HS: Writing – review & editing.
